# Notes and Formulæ

**Published:** 1889-04

**Authors:** 


### PRACTICAL NOTES AND FORMULÆ.

**Rheumatism.**—Dr. Merrick says in *Med. Brief.*—Here is a prescription which seems to have a good effect on many cases of rheumatism:

| | | |
|---|---|---|
| 64-1 | Salicylate of Sodæ, | 3.32 |
| | Pulv. Ipecac Comp. | 1.32 |
| | Quin. Sulph. | .32 |
| | Sodæ Bicarb. | .32 |
| | Pul. Acaciæ, | .16 |
| | Fl. Ext. Cascara Sagrada. | 1.00 |

The above ingredients will make about sixty-four pills, the figures express the amount in each pill. Now, if four pills are ordered at a dose the symbol might be changed to 16–¼.

**Lithium and Arsenic in Diabetes.**—Dr. Vigier recommends the following formula for the treatment of diabetes:

| | | |
|---|---|---|
| ℞. | Carbonate of lithium, | 1½ grs. |
| | Arseniate of sodium, | $\frac{1}{20}$ gr. |

For one pill. Take two a day.—*Ex.*

**Cancer.**—Decided results in the treatment of cancer have been obtained with the following preparation:

| | | |
|---|---|---|
| ℞. | Creasoti Puri. | 5 drachms. |
| | Sodii Bicarb. | 5 drachms. |
| | Ol. Morrhuæ, | 5 drachms. |

M. Put in 100 gelatine capsules. Sig.: Three capsules three times daily, after each meal.—*Ib.*

**New Preserving Fluid.**—

| | | | |
|---|---|---|---|
| ℞. | Water, | 35 | ounces. |
| | Common Salt, | 3 | ounces. |
| | Saltpetre, | 6½ | drachms. |
| | Carbolic Acid, | 1¼ | drachms. |
| | Glycerine, | 4 | drachms. |
| | Amylic Alcohol, | 1¾ | drachms. |
| | Or. Ethylic Alcohol, | 3½ | ounces. |

Specimens should be first soaked in a strong brine and then placed in a large quantity of this fluid.—*Ib.*

**For Ringworm.**—A saturated solution of salicylic acid in collodion is a prompt cure for ringworm. The solution is painted on the affected parts once a day. One application generally suffices.—*Ib.*

**Chilblains.**—

| | | |
|---|---|---|
| ℞. | Flex. Collodion. . . . . . | 4 drachms. |
| | Ol. Ricini. . . . . . | 4 drachms. |
| | Spts. Terebinth. . . . . . | 4 drachms. |

M. Sig.: To be used twice or thrice daily.—*Ib.*

**Pomade for Antiseptic Dressing**—

| | |
|---|---|
| Iodoform, . . . . | ʒ ss |
| Essence of Eucalyptus, . . | ʒ iv |
| Paraffine, . . . . . | |
| Vaseline, . . . . . | ää ℥jss |

**Eczema Marginatum**, or more properly ringworm of the thighs, is an annoying trouble which fortunately promptly yields to treatment. The best application which I have found so far in this form of tinea corporis is the following:

| | | |
|---|---|---|
| ℞ | Hydrargyri bihclorodi . | gr. iss. |
| | Tinct. benzoini comp. . | ℥j. |

M. Fiat solutio.
Sig. Paint on affected part once daily.

It frequently occurs that two or three applications are sufficient to bring about complete relief.—*Med. Chips.*

The following is said to be available in albuminaria of pregnancy:

| | | |
|---|---|---|
| ℞. | Acid benzoic. . . . . . | gr. v |
| | Potassii bicarb. . . . . . | gr. xxv |
| | Spirit chloroform. . . . . . | m. v. |
| | Syrup simplicis, . . . . . | f. ʒss |
| | Ag. destil, q. s. . . . . . | ℥ss |

M. Sig.—Every two hours.

In cases of chronic interstitial nephritis, Da Costa advises a diet of milk, fish, an occasional laxative of Rochelle Salts, and,

| | |
|---|---|
| Caffeinæ. . . . . . . . | |
| Sodii salicylate aa. . . . . . | gr. iii |
| Syrup aurantii . . . . . . | |
| Aq. destil, ad. . . . . . . | f ʒ iv |

S.—At one dose.—*Ib.*

